# Clinical characteristics and risk factors for poor prognosis of RSV lower respiratory tract infection: a two-center study

**DOI:** 10.1186/s12887-026-07480-4

**Published:** 2026-08-10

**Authors:** Tianyu Chen, Xiaolei Guo, Wenyin Tang, Feng Liu, Jiahua Jv, Hang Sun

**Affiliations:** 1 Department of Respiration, Children’s Hospital of Nanjing Medical University, Nanjing, Jiangsu China; 2 Department of Pediatrics, Gaoyou People’s Hospital, Gaoyou, Jiangsu China; 3 Department of Emergency, Children’s Hospital, Zhejiang University School of Medicine, National Clinical Research Center for Children and Adolescents’ Health and Diseases, Hangzhou, Zhejiang China; 4 Department of Clinical Laboratory, Children’s Hospital of Nanjing Medical University, Nanjing, Jiangsu China

**Keywords:** Respiratory syncytial virus, Lower respiratory tract infection, Poor prognosis, Risk factors

## Abstract

**Objective:**

Respiratory syncytial virus (RSV) is a leading cause of acute lower respiratory tract infection (LRTI) in children under five years of age. In this study, we investigated the value of simple and readily available clinical indicators in predicting poor prognosis in children with RSV-LRTI.

**Methods:**

This was a retrospective cohort study of children with RSV-LRTI admitted to Children’s Hospital of Nanjing Medical University (April 2023–May 2024) and Gaoyou People’s Hospital (December 2024–June 2025). Demographic and clinical data were collected from patient records, and patients were stratified based on three adverse prognostic events: admission to Intensive Care Unit (ICU), prolonged hospital stay, and severe pneumonia.

**Results:**

A total of 257 eligible children were enrolled, of whom 254 (98.8%) exhibited cough, 144 (56.0%) presented with wheezing and 194 (75.4%) developed febrile symptoms. 220 (85.6%) cases of RSV-LRTI occurred in winter and spring, with a higher proportion of cases occurring in infants under 1 year of age. A higher respiratory rate at admission was independently associated with ICU admission (Firth regression: OR 1.132, both *P* < 0.05), though this finding was limited by the small number of ICU cases. The presence of three concave sign was independently associated with prolonged hospital stay (OR 5.242, 95% CI 1.360-19.951, *P* = 0.017). Procalcitonin (PCT) was an independent risk factor for severe pneumonia (OR 1.616, 95% CI 1.074–2.837, *P* = 0.020).

**Conclusion:**

Higher respiratory rate at admission was independently associated with ICU admission, and the presence of three concave sign was independently associated with prolonged hospital stay. PCT was an independent risk factor for severe pneumonia. These simple clinical indicators facilitate early identification of children at risk of adverse outcomes.

**Supplementary Information:**

The online version contains supplementary material available at https://doi.org/10.1186/s12887-026-07480-4.

## Introduction

Respiratory syncytial virus (RSV) is the leading pathogen causing acute lower respiratory tract infection (LRTI) in children under 5 years of age, and nearly all children experience at least one RSV infection by the age of 2 years [1]. A global study in 2019 estimated that RSV was associated with approximately 33 million episodes of acute LRTI worldwide, among which 3.6 million required hospitalization, and an estimated 66,000 to 190,000 deaths occurred in children under 5 years, with 95% of these deaths happening in low- and middle-income countries and significant regional epidemiological differences [1]. Approximately 22% of children with severe RSV infection require admission to an intensive care unit (ICU) [2]. China bears one of the heaviest RSV-related disease burdens globally. According to statistics, in 2019, RSV caused about 3.5 million episodes of acute LRTI in Chinese children under 5 years, accounting for more than 10% of the global total [3].

RSV infection in infants and young children commonly causes bronchiolitis and pneumonia, presenting with fever, cough, wheezing, three concave sign (refers to suprasternal, intercostal, and subcostal retractions), and hypoxemia; some may experience apnea [4]. It can lead to long-term respiratory sequelae, increasing the risk of recurrent wheezing, asthma, and impaired lung function [5].Therefore, identifying risk factors for poor prognosis in RSV-LRTI is clinically important. Known risk factors include prematurity, congenital heart disease, passive smoking, tachypnea, lack of breastfeeding, low birth weight, and neonatal intensive care history. A systematic review and meta-analysis by Shi et al. identified potential variables for prediction models [6], and Wrotek et al. found that blood gas analysis could predict the need for intensive care [7]. Timely identification and early intervention of poor prognosis are particularly important for RSV infection. However, relevant studies in China remain limited, and clinicians often have to rely on empirical judgment. Therefore, it is necessary to thoroughly investigate the risk factors for poor prognosis in RSV-LRTI.

To early detect adverse outcomes in RSV infection, we conduct a two-center retrospective clinical cohort study to elucidate the characteristics of RSV-LRTI, providing a reference for appropriate management in clinical practice and effectively reducing the rate of severe disease.

## Materials and methods

### Research design

**Fig. 1:**
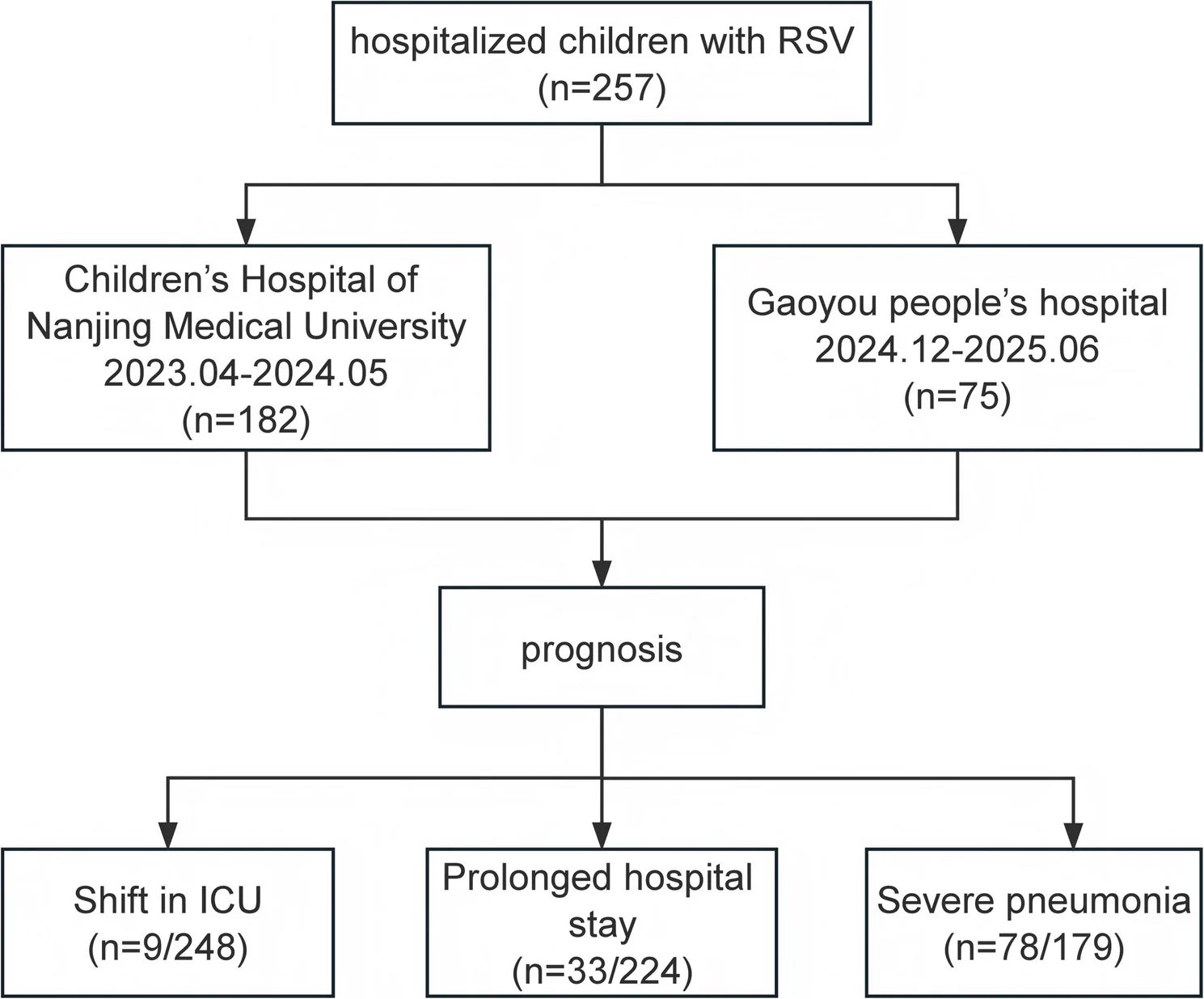
Flowchart of the study. RSV: respiratory syncytial virus; ICU: Intensive Care Unit

We collected data from children hospitalized for LRTI caused by RSV with complete clinical records at the Department of Respiratory Medicine, Children’s Hospital of Nanjing Medical University (April 2023–May 2024) and at Gaoyou People’s Hospital (December 2024–June 2025). Patients diagnosed with RSV-LRTI at admission were enrolled in a retrospective cohort, and their clinical data were collected. They were stratified based on three adverse outcomes: admission to ICU, prolonged hospital stay and severe pneumonia. The study flow chart is shown in Fig. 1. This study was approved by the Ethics Committees of Children’s Hospital of Nanjing Medical University and Gaoyou People’s Hospital (ethics approval numbers: 202402017-1, LLKY-2024-10-02).

### Inclusion criteria and exclusion criteria

Children meeting all the following criteria were included in the cohort: (a) Age between 28 days and 10 years; (b) Positive RSV nucleic acid test by real-time fluorescence quantitative Polymerase Chain Reaction (PCR) from nasopharyngeal swabs; (c) Chest imaging findings suggest bronchitis, bronchiolitis, or bronchopneumonia; (d) Complete clinical data.

Meeting any one of criteria a, b, or c led to exclusion: (a) Evidence of coinfection with other pathogens during the disease course, including positive Mycoplasma pneumoniae nucleic acid in sputum or bronchoalveolar lavage fluid, positive culture of sputum or bronchoalveolar lavage fluid, or positive nucleic acid test for other viruses in throat swabs; (b) Diagnosis of pneumonia within 28 days before admission and still in the recovery phase; (c) Underlying diseases such as bronchial asthma, congenital bronchopulmonary dysplasia, bronchial malformation, or congenital heart disease.

### Clinical data

To facilitate early identification of children with poor prognosis of RSV infection and to ensure applicability to both primary community hospitals and higher-level hospitals, the clinical data collected in this study comprised common clinical manifestations and routine rapid test results that can be routinely performed in primary community hospitals. Co-infection screening was performed systematically for all patients, including throat swabs for PCR of other respiratory viruses, mycoplasma and chlamydia nucleic acid, and sputum were tested for bacterial culture.

It should be noted that while our study employed real-time fluorescence quantitative PCR for RSV confirmation due to its high sensitivity and specificity as the reference standard, rapid antigen detection tests are more commonly available in primary care settings. Rapid antigen tests have a sensitivity of approximately 80–90% in children with moderate-to-severe LRTI and a high specificity (> 95%), making them a practical alternative for confirming RSV etiology during peak seasons. Importantly, none of the prognostic risk factors identified in this study depends on the specific method used for RSV diagnosis; therefore, our findings are directly applicable to patients diagnosed by either PCR or rapid antigen testing.

### Clinical prognosis

In-hospital prognostic indicators included length of hospital stay, hospitalization costs, prolonged hospital stay, admission to ICU, and diagnosis of severe pneumonia. Hospital stay exceeding the 75th percentile (> 7 days) was defined as prolonged hospital stay. In this study cohort, there were 33 patients with a hospital stay of more than 7 days, accounting for 12.8% of the total cohort.

Admission to ICU was defined as transfer from the general ward to the pediatric ICU (PICU). The decision followed objective criteria uniformly applied in both centers, based on Expert Consensus on Clinical Early Warning and Early Decision-Making for Severe Pneumonia in Children (2023) [8]: (1) Moaning or altered consciousness; (2) Tachypnea (excluding effects of fever or crying; ≥70 breaths/min in infants, ≥ 50 breaths/min in older children) with respiratory distress; (3) Frequent apneas or slow, irregular breathing, with or without elevated PaCO₂; (4) Severe hypoxemia (sea-level SaO₂ <0.90 or PaO₂/FiO₂ <26.6 kPa); (5) Signs of shock or cardiac insufficiency; (6) Pediatric early warning score > 6. To ensure consistency between the two centers, we retrospectively reviewed medical records of all ICU-admitted patients and confirmed that all admissions met these criteria and that no patient was admitted for non-clinical reasons. The same criteria were used in both hospitals. Respiratory support modalities were available only in the PICU, with identical protocols across centers.

Severe pneumonia was defined according to the Guideline for Diagnosis and Treatment of Community-Acquired Pneumonia in Children (2019 Revision) issued by the Chinese National Health Commission [9]. According to this guideline, severe pneumonia was defined as meeting any one of the following criteria: (1) poor general condition; (2) hyperpyrexia or persistent high fever for > 5 days; (3) dehydration, refusal to eat, or altered mental status; (4) hypoxemia: respiratory rate ≥ 70/min in infants or ≥ 50/min in children > 1 year of age; presence of cyanosis or groaning, nasal flaring, inspiratory chest depression; intermittent apnea or oxygen saturation < 92%; (5) presence of extrapulmonary complications; (6) chest X-ray or CT showing ≥ 2/3 unilateral lung infiltration, multilobar infiltration, pleural effusion, pneumothorax, atelectasis, or lung necrosis.

### Statistical analysis

Statistical analyses were performed using SPSS 27.0, R language version 4.5.2, and GraphPad Prism 8. A clinical database of children with RSV-LRTI was established using SPSS 27.0. All collected data were initially screened for missing values. When missing data were detected, the type and distribution of missingness were visualized. To maximize the use of collected data and avoid the impact of missing values on the results, given that the proportion of missing data was < 15%, we performed multiple imputation [10]. Epidemiological characteristics and age distribution analyses were conducted using R language version 4.5.2.

Univariate analysis was performed on the imputed dataset. For continuous variables, normality was tested: normally distributed data were analyzed using independent samples t-tests, otherwise, Mann-Whitney U tests; results were presented as interquartile range (IQR). For binary variables, chi-square or Fisher’s exact test was used, with data presented as counts (%). Variables with *P* < 0.05 were considered significant.

Candidate variables for multivariable analysis were those with *P* < 0.05 in univariate analysis. Variables with complete or quasi-complete separation (zero events in one outcome category) were excluded to avoid estimation failure. Due to the limited number of events for outcomes and to ensure consistent handling of potential overfitting and separation, we used Firth’s penalized logistic regression as the primary analytic method for all multivariable models. For transparency, the corresponding standard binary logistic regression results are provided in the supplementary materials. Variables with *P* < 0.05 and odds ratios (ORs) with 95% confidence intervals (CIs) > 1 were considered statistically significant independent risk factors. Borderline or non-significant variables with potential clinical importance should be interpreted cautiously and validated in future larger studies.

Sensitivity analysis with binary tachypnea. To address the age-dependency of respiratory rate, we repeated the multivariable Firth regression models using a binary indicator of age-specific tachypnea (defined according to APLS/PALS guidelines [11]) in place of the continuous respiratory rate. We also calculated VIF for all candidate variables in the ICU model to assess multicollinearity.

## Results

### General clinical characteristics

From April 2023 to May 2024, a total of 182 children with RSV-LRTI who met the inclusion and exclusion criteria were admitted to the Department of Respiratory Medicine, Children’s Hospital of Nanjing Medical University. From December 2024 to June 2025, 75 eligible children were admitted to Gaoyou People’s Hospital. Overall, 257 children were enrolled, including 140 males (54.4%) and 117 females (45.5%). The mean age was 23.28 months, and 153 children (59.5%) were aged ≤ 2 years. Regarding clinical features, 194 (75.4%) presented with fever, 254 (98.9%) with cough, and 144 (56.0%) with wheezing. Physical examination revealed three concave sign in 20 (7.7%), moist rales in 120 (46.6%), and wheezing sound in 134 (52.1%). Medical history showed that 21 (8.1%) were premature, 60 (23.3%) had a history of personal allergens, and 25 (9.7%) had a history of wheezing. In terms of outcomes, 9 (3.5%) required ICU admission, 33 (12.8%) had prolonged hospital stay, and 78 (30.3%) developed severe pneumonia (Table 1). Among the 9 children admitted to the ICU, 8 (4.4% of the tertiary center cohort) and 1 (1.3% of the county hospital cohort) met the pre-defined respiratory failure criteria. No significant inter-center difference in ICU admission thresholds was identified upon chart review. The two centers of pediatric patients exhibited differences in hospitalization duration, hospitalization costs, prolonged hospital stays, and severe pneumonia outcomes (Supplementary Figure S1).

**Table 1:** Demographic data and general clinical data of pediatric patients

Characteristics	Total (*n* = 257)	Children’s Hospital of Nanjing Medical University (*n* = 182)	Gaoyou people’s Hospital (*n* = 75)	*P* value
Sex (n, %)				**0.001**
Male	140 (54.5%)	111 (61.0%)	29 (38.7%)	
Female	117 (45.5%)	71 (39.0%)	46 (61.3%)	
Age (y)				**0.001**
≤ 2y	153 (59.5%)	120 (65.9%)	33 (44.0%)	
>2y	104 (40.5%)	62 (34.1%)	42 (56.0%)	
Clinical Characteristics				
Disease in winter and spring	220 (85.6%)	146 (80.2%)	74 (98.7%)	**< 0.001**
Cough	254 (98.8%)	181 (99.5%)	73 (97.3%)	0.205
Wheeze	144 (56.0%)	116 (63.7%)	28 (37.3%)	**< 0.001**
Fever	194 (75.5%)	142 (78.0%)	52 (69.3%)	0.153
Three concave sign	20 (7.8%)	12 (6.6%)	8 (10.7%)	0.307
Wheezing sound	134 (52.1%)	97 (53.3%)	37 (49.3%)	0.585
Moist Rales	120 (46.7%)	92 (50.5%)	28 (37.3%)	0.056
Bronchopneumonia	209 (81.3%)	150 (82.4%)	59 (78.7%)	0.598
Past history				
Preterm infant	21 (8.2%)	20 (11.0%)	1 (1.3%)	**0.010**
Breastfeeding	127 (49.4%)	79 (43.4%)	48 (64.0%)	**0.004**
Personal allergen history	60 (23.3%)	35 (19.2%)	25 (33.3%)	**0.022**
Wheezing history	25 (9.7%)	14 (7.7%)	11 (14.7%)	0.105
Poor prognosis				
ICU admission	9 (3.5%)	8 (4.4%)	1 (1.3%)	0.400
Prolonged hospital stay	33 (12.8%)	15 (8.2%)	18 (24.0%)	**0.001**
Severe pneumonia	77 (30.0%)	76 (41.8%)	1 (1.3%)	**< 0.001**

Bold values indicate statistical significance (*P*< 0.05)

Baseline characteristics of the two centers are compared in Table 1. Compared with the Gaoyou cohort, the Nanjing cohort had a higher proportion of infants aged ≤ 2 years (65.9% vs. 44.0%, *P* = 0.001), wheezing (63.7% vs. 37.3%, *P* < 0.001), and severe pneumonia (41.8% vs. 1.3%, *P* < 0.001), as well as a higher rate of preterm birth (11.0% vs. 1.3%, *P* = 0.010). In contrast, the Gaoyou cohort had a higher proportion of breastfeeding (64.0% vs. 43.4%, *P* = 0.004) and personal allergen history (33.3% vs. 19.2%, *P* = 0.022). These differences reflect the distinct roles of the two institutions (a tertiary referral center vs. a county-level hospital) in the regional healthcare system. Despite these differences, the association directions of the primary predictors remained consistent between the Nanjing cohort and the combined cohort (Supplementary Table S6); however, the Gaoyou cohort was not analyzed due to its very limited number of ICU admissions (*n* = 1) and severe cases (*n* = 1).

### Epidemiological characteristics

**Fig. 2:**
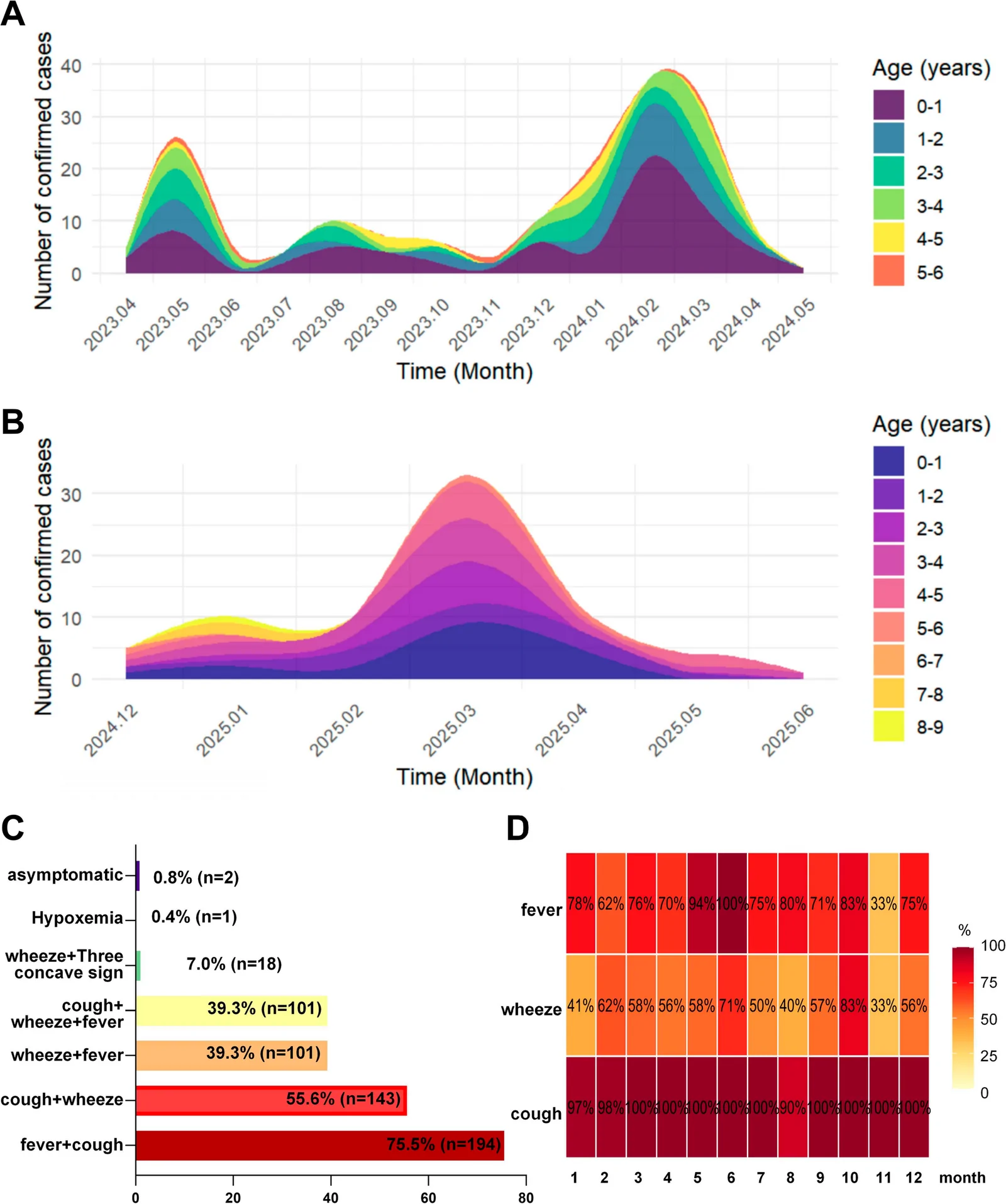
Seasonal prevalence of RSV, age of children, and symptoms. **A** Temporal distribution and age of RSV-infected children at Children’s Hospital of Nanjing Medical University from April 2023 to May 2024. **B** Temporal distribution and age of RSV-infected children at Gaoyou People’s Hospital from December 2024 to June 2025. **C** The number and proportion of children with different symptom combinations. Categories are not mutually exclusive; each child may be counted in multiple categories. **D** The proportion of cough, fever, and wheezing occurring in the child across different months

RSV-LRTI was detected throughout the year. Among Children’s Hospital of Nanjing Medical University, 20 cases (11.0%) were detected in summer (June–August 2023), 16 cases (8.8%) in autumn (August–November 2023), 70 cases (38.5%) in winter (December 2023–February 2024), and 45 cases (24.7%) in spring (March–May 2024). At Gaoyou People’s Hospital, 25 cases (33.3%) were detected in winter (December 2024–February 2025), and 49 cases (65.3%) in spring (March–May 2025). RSV epidemics occurred in late winter and early spring, and infants under 1 year of age were the main population affected by LRTI (Fig. 2A-B). Most children presented with fever, cough, and wheezing (Fig. 2C and Supplementary Figure S2). Specifically, 101 cases (39.3%) exhibited all three symptoms simultaneously. Notably, all children who had both wheeze and fever also had cough, making the “wheeze+fever” group identical to the three-symptom group. Additionally, 18 cases (7.0%) presented with both wheezing and a positive three-concave sign; two cases were asymptomatic, and one case developed hypoxemia (oxygen saturation at admission = 90%). No significant differences were observed in the incidence rates of wheezing, cough, and fever across months, indicating that these symptoms in pediatric patients are largely independent of seasonal variations (Fig. 2D).

### Risk factors for ICU admission in children with RSV-LRTI

Children were divided into an ICU admission group (9 cases) and a non-ICU group (248 cases) based on whether they required ICU care. Comparison of clinical data showed that children in the ICU group had a higher respiratory rate at admission (43 breaths/min, IQR 40–47), lower oxygen saturation at admission (96%, IQR 94%–98%), and a higher proportion of three concave sign (7 cases, 77.8%). They also exhibited higher systemic inflammatory levels: PCT 0.12 (0.08–0.35) ng/ml and CRP 5.0 (0.6–11.9) mg/L. All differences were statistically significant (all *P* < 0.05) (Table 2).

**Table 2:** Univariate analysis of ICU admission

Characteristics	Non-ICU (*n* = 248)	ICU (*n* = 9)	*P* value
Sex (n, %)			0.276
Male	133 (53.6%)	7 (77.8%)	
Female	115 (46.4%)	2 (22.2%)	
Age (month)	18.5 (6.0–39.0)	18.0 (7.0–32.0)	0.268
Wheeze (n, %)	136 (54.8%)	8 (88.9%)	0.093
Cough (n, %)	245 (98.8%)	9 (100.0%)	1.000
Fever (n, %)	187 (75.4%)	7 (77.8%)	1.000
Pre-admission fever days (d)	2.0 (0–4.0)	4.0 (1.0–6.0)	0.209
Respiratory rate (times/min)	30 (28–32)	43 (40–47)	**< 0.001**
Oxyhemoglobin saturation (%)	98 (97–99)	96 (94–98)	**0.034**
Three concave sign (n, %)	13 (5.2%)	7 (77.8%)	**< 0.001**
Wheezing sound (n, %)	130 (52.4%)	4 (44.4%)	0.896
Moist Rales (n, %)	115 (46.4%)	5 (55.6%)	0.840
Preterm infant (n, %)	20 (8.1%)	1 (11.1%)	1.000
Personal allergen history (n, %)	58 (23.4%)	2 (22.2%)	1.000
Wheezing history (n, %)	25 (10.1%)	0 (0.0%)	0.667
Bronchopneumonia (n, %)	201 (81.0%)	8 (88.9%)	0.975
PCT (ng/ml)	0.10 (0.06–0.18)	0.12 (0.08–0.35)	**0.039**
CRP (mg/L)	2.7 (0.5–10.6)	5.0 (0.6–11.9)	**0.015**
WBC (×10^9^/L)	8.0 (6.1–10.9)	9.8 (7.0-12.7)	0.193
Neutrophils (%)	42.5 (25.5–57.4)	47.4 (28.4–61.9)	0.086
Lymphocyte (%)	47.3 (32.9–63.1)	41.9 (28.1–59.3)	0.059
L/N	1.1 (0.6–2.4)	0.4 (0.3–1.7)	0.080
ALT (U/L)	18.0 (15.0–24.0)	18.0 (14.0–24.0)	0.141
AST (U/L)	38.0 (31.0-45.5)	35.0 (30.0–46.0)	0.322
LDH (U/L)	312.0 (276.5-373.1)	337.0 (290.0-396.0)	0.212
CK (U/L)	89.0 (63.0-125.5)	70.0 (50.0-147.0)	0.093

Bold values indicate statistical significance (*P*< 0.05)

**Fig. 3:**
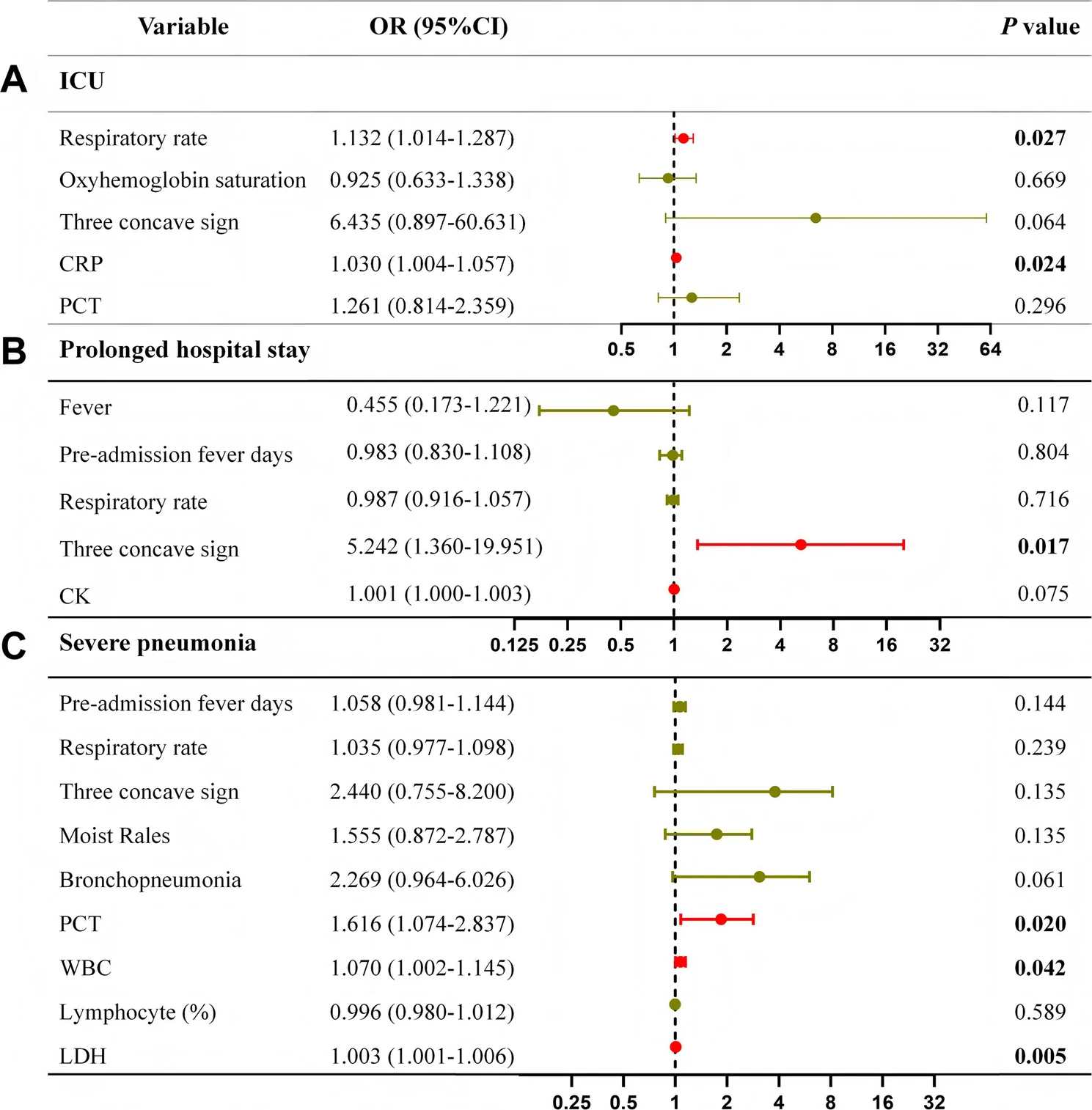
Firth’s penalized logistic regression analysis of several prognoses for RSV. **A** Firth regression analysis for ICU admission. **B** Risk factor of prolonged hospital stay. **C** Firth regression analysis of severe pneumonia

Due to the limited number of ICU events (*n* = 9), standard logistic regression is prone to overfitting. Therefore, we used Firth’s penalized logistic regression as the primary analysis. As shown in Fig. 3A, higher respiratory rate at admission (OR 1.132, 95% CI 1.014–1.287, *P* = 0.027) and higher CRP (OR 1.030, 95% CI 1.004–1.057, *P* = 0.024) were statistically associated with ICU admission. Three concave sign showed a trend toward increased risk but did not reach statistical significance (OR 6.435, 95% CI 0.897–60.631, *P* = 0.064). Oxyhemoglobin saturation and PCT were not significant. The wide confidence intervals reflect the small number of events; these results should be considered exploratory. The corresponding standard logistic regression (Supplementary Figure S3A) yielded directionally similar but less stable estimates.

As a sensitivity analysis, we replaced continuous respiratory rate with age-specific tachypnea (binary) in the Firth regression model (Supplementary Table S1-S3). Although tachypnea was more common in the ICU group (33.3% vs. 1.6%, *P* < 0.001), it did not remain independently significant in the multivariable model (OR 2.978, 95% CI 0.338–22.706, *P* = 0.316), likely due to loss of statistical power from dichotomization and the very small number of ICU events.

### Risk factors for prolonged hospital stay in children with RSV-LRTI

Children were divided into a prolonged stay group (33 cases) and a non-prolonged group (224 cases) based on whether the length of hospital stay exceeded the 75th percentile of the total cohort (> 7 days). In the non-prolonged group, all children had cough (224, 100.0%), 174 (77.7%) had fever, and the median pre-admission fever days was 3.0 (1.0–5.0) days. In the prolonged stay group, 30 (90.9%) had fever, 20 (60.6%) had cough, and the median pre-admission fever days was 2.0 (0.0–4.0) days. The prolonged stay group had a respiratory rate at admission of 30.0 (28.0–36.0) breaths/min, 7 cases (21.2%) with three concave sign, and a CK level of 118.0 (81.0–188.0) U/L. In contrast, children with a hospital stay ≤ 7 days had a median respiratory rate of 30.0 (28.0–32.0) breaths/min, 13 cases (5.8%) with an inspiratory three concave sign, and a median CK of 79.0 (52.0–120.0) U/L. Overall, compared with the non-prolonged group, the prolonged stay group had a higher respiratory rate at admission, a higher proportion of positive three concave sign, and higher CK levels, indicating more severe illness at admission (all *P* < 0.05) (Table 3).

**Table 3:** Univariate analysis of prolonged hospital stay

Characteristics	(*n* = 224)	Prolonged hospital stay (*n* = 33)	*P* value
Sex (n, %)			0.462
Male	120 (53.6%)	20 (60.6%)	
Female	104 (46.4.%)	13 (39.4%)	
Age (month)	19.5 (6.5–38.5)	13.0 (5.0–31.0)	0.149
Wheeze (n, %)	122 (54.5%)	22 (66.7%)	0.196
Cough (n, %)	224 (100.0%)	30 (90.9%)	**< 0.001**
Fever (n, %)	174 (77.7%)	20 (60.6%)	**0.049**
Pre-admission fever days (d)	3.0 (1.0–5.0)	2.0 (0.0–4.0)	**0.031**
Respiratory rate (times/min)	30.0 (28.0–32.0)	30.0 (28.0–36.0)	**0.028**
Oxyhemoglobin saturation (%)	98.0 (97.0–99.0)	98.0 (96.0–99.0)	0.136
Three concave sign (n, %)	13 (5.8%)	7 (21.2%)	**0.006**
Wheezing sound (n, %)	114 (50.9%)	20 (60.6%)	0.352
Moist Rales (n, %)	107 (47.8%)	13 (39.4%)	0.456
Preterm infant (n, %)	19 (8.5%)	2 (6.1%)	0.894
Personal allergen history (n, %)	54 (24.1%)	6 (18.2%)	0.517
Wheezing history (n, %)	23 (10.3%)	2 (6.1%)	0.655
Bronchopneumonia (n, %)	181 (80.8%)	28 (84.8%)	0.643
PCT (ng/ml)	0.1 (0.06–0.2)	0.1 (0.06–0.3)	0.482
CRP (mg/L)	3.4 (0.5–11.1)	2.7 (0.9–10.2)	0.422
WBC (×10^9^/L)	8.7 (6.5–11.5)	8.1 (6.5–9.5)	0.335
Neutrophils (%)	43.4 (25.8–58.5)	43.3 (30.8–62.2)	0.230
Lymphocyte (%)	46.3 (31.8–62.6)	44.0 (27.4–55.5)	0.275
L/N	1.1 (0.5–2.4)	1.0 (0.4–1.7)	0.223
ALT (U/L)	18.0 (14.0–24.0)	20.0 (17.0–23.0)	0.124
AST (U/L)	37.0 (30.0–45.0)	38.0 (32.0–47.0)	0.136
LDH (U/L)	316.5 (279.0-382.0)	310.0 (283.0-370.0)	0.415
CK (U/L)	79.0 (52.0-120.0)	118.0 (81.0-188.0)	**< 0.001**

Bold values indicate statistical significance (*P*< 0.05)

Because cough exhibited near-complete separation (100% vs. 90.9%) and lacked clinical discriminative value (98.8% overall prevalence), it was excluded from multivariable analysis. Firth’s penalized logistic regression was then performed on the remaining five variables (fever, pre-admission fever days, respiratory rate, three concave sign, and CK) to address potential overfitting. As shown in Fig. 3B, three concave sign was the only independent risk factor for prolonged hospital stay (OR = 5.242, 95% CI: 1.360-19.951, *P* = 0.017). None of the other variables reached statistical significance. The variance inflation factors among all independent variables were all less than 5, indicating no multicollinearity among them (Supplementary Table S4). Full results including exploratory models (standard logistic regression and Firth regression with the cough variable) are presented in Supplementary Figure S3B and Supplementary Table S5.

### Risk factors for severe pneumonia in children with RSV-LRTI

According to the diagnostic criteria for severe pneumonia, enrolled children were divided into a severe pneumonia group (77 cases) and a non-severe pneumonia group (180 cases). In the severe pneumonia group, the median pre-admission fever days was 4.0 (1.0–6.0) days, the respiratory rate at admission was 30.0 (28.0–34.0) breaths/min, 12 (15.6%) had positive inspiratory three concave sign, 45 (58.4%) had crackles on auscultation, and 70 (90.9%) had pneumonia on chest imaging. PCT levels in the severe pneumonia group were not higher in central tendency (median 0.10 ng/mL in both groups), but a greater proportion of severe cases had values in the upper range (IQR 0.08–0.40 vs. 0.06–0.20 ng/mL), contributing to the statistical association. Compared with the mild pneumonia group, the severe pneumonia group had higher levels of WBC (9.8 × 10⁹/L, IQR 7.0–12.7), and LDH (337.0 U/L, IQR 290.0–396.0), but a lower lymphocyte percentage (47.4%, IQR 28.4%–61.9%); all differences were statistically significant (*P* < 0.05) (Table 4). Multivariate logistic regression analysis identified PCT, WBC, and LDH as independent risk factors for severe pneumonia, with ORs of 1.749 (95% CI: 1.055–2.900, *P* = 0.030), 1.074 (95% CI: 1.003–1.149, *P* = 0.040), and 1.004 (95% CI: 1.001–1.006, *P* = 0.006), respectively (Fig. 3C).

**Table 4:** Univariate analysis of severe pneumonia

Characteristics	(*n* = 179)	Severe pneumonia (*n* = 78)	*P* value
Sex (n, %)			0.416
Male	95 (52.8%)	45 (58.4%)	
Female	85 (47.2.%)	32 (41.6%)	
Age (month)	18.5 (6.0–39.0)	18.0 (7.0–32.0)	0.334
Wheeze (n, %)	95 (52.8%)	49 (63.6%)	0.131
Cough (n, %)	178 (98.9%)	76 (98.7%)	1.000
Fever (n, %)	130 (72.2%)	64 (83.1%)	0.081
Pre-admission fever days (d)	2.0 (0.0–4.0)	4.0 (10.-6.0)	**0.001**
Respiratory rate (times/min)	30.0 (28.0–32.0)	30.0 (28.0–34.0)	**0.010**
Oxyhemoglobin saturation (%)	98.0 (97.0–99.0)	98.0 (96.0-100.0)	0.451
Three concave sign (n, %)	8 (4.4%)	12 (15.6%)	**0.004**
Wheezing sound (n, %)	95 (52.8%)	39 (50.6%)	0.786
Moist Rales (n, %)	75 (41.7%)	45 (58.4%)	**0.015**
Preterm infant (n, %)	12 (6.7%)	9 (11.7%)	0.214
Personal allergen history (n, %)	47 (26.1%)	13 (16.9%)	0.147
Wheezing history (n, %)	16 (8.9%)	9 (11.7%)	0.496
Bronchopneumonia (n, %)	139 (77.2%)	70 (90.9%)	**0.014**
PCT (ng/ml)	0.1 (0.06–0.2)	0.1 (0.08–0.4)	**0.006**
CRP (mg/L)	2.7 (0.5–10.6)	5.0 (0.6–11.9)	0.068
WBC (×10^9^/L)	8.0 (6.1–10.9)	9.8 (7.0-12.7)	**0.004**
Neutrophils (%)	42.5 (25.5–57.4)	47.4 (28.4–61.9)	0.158
Lymphocyte (%)	47.3 (32.9–63.1)	41.9 (28.1–59.3)	**0.049**
L/N	1.1 (0.6–2.5)	0.9 (0.5–2.2)	0.140
ALT (U/L)	18.0 (15.0–24.0)	18.0 (14.0–24.0)	0.336
AST (U/L)	38.0 (31.0-45.5)	35.0 (30.0–46.0)	0.153
LDH (U/L)	312.0 (276.5-373.1)	337.0 (290.0-396.0)	**0.021**
CK (U/L)	89.0 (63.0-125.5)	70.0 (50.0-147.0)	0.107

Bold values indicate statistical significance (*P*< 0.05)

Firth’s penalized logistic regression identified PCT (OR 1.616, 95% CI 1.074–2.837, *P* = 0.020), WBC (OR 1.070, 95% CI 1.002–1.145, *P* = 0.042), and LDH (OR 1.003, 95% CI 1.001–1.006, *P* = 0.005) as independent risk factors for severe pneumonia. The results were consistent with standard logistic regression (Supplementary Figure S3C), and Firth regression confirmed robustness.

## Discussion

In this two-center study, we retrospectively collected clinical data from 257 pediatric patients with RSV-LRTI. RSV exhibited seasonal prevalence, with higher incidence during winter and spring. Among RSV-LRTI patients, 99.2% presented with at least one symptom (cough, fever, or wheezing) during the disease course, and 81.3% demonstrated pneumonia on imaging findings.

RSV frequently occurs in the first year of life, confirmed by our peak incidence in infants, likely due to low immunity and immature small airways [12]. Although prematurity and low birth weight are known risk factors [13], prematurity was not significantly associated with any adverse outcome in our study. However, this finding should be interpreted with caution due to the small number of preterm infants and the exclusion of children with bronchopulmonary dysplasia and other chronic respiratory comorbidities. Thus, our results apply only to preterm infants without chronic respiratory sequelae. RSV epidemiology correlates with temperature and humidity. Northern China has winter-spring peaks, while southern China may have prolonged seasons due to the humid climate [14]. The winter-spring peak of RSV-LRTI in both centers did not coincide with the summer rainy season, consistent with the known epidemiology of RSV in temperate regions. During COVID-19, non-pharmaceutical interventions delayed RSV circulation, followed by an “immunity debt” after lifting measures [15]. Our study showed an annual prevalence with a winter-spring predominance, but infections also occurred in the summer of 2023, possibly reflecting post-intervention off-season resurgence and individual immune status [16].

Multivariate regression identified higher admission respiratory rate and CRP as independent PICU risk factors. Previous studies have shown that a marked increase in respiratory rate is closely related to the severity of respiratory distress in children with RSV infection [17]. Those with a rate > 70 breaths/min had a significantly increased risk of ICU admission [18]. However, measurement can be affected by crying or fever and should be combined with three concave sign and wheezing. We also performed sensitivity analyses using age-specific tachypnea instead of continuous respiratory rate. Although tachypnea was more frequent in the ICU group, it was not independently significant in the multivariable model, likely due to loss of power from dichotomization and the small event number. Continuous respiratory rate remains our primary predictor. However, the small effect sizes and the limited number of ICU events suggest that these findings are exploratory and should not be overinterpreted for clinical decision-making. A multimodal clinical assessment remains essential. CRP elevation in our cohort may reflect a virus-directed inflammatory response or, in some cases, an undetected bacterial co-infection, despite systematic screening [19]. However, given the small effect size, CRP should be interpreted cautiously as a supportive indicator rather than a robust independent predictor for ICU admission. Our definition of ICU aligns with international standards on key severity indicators, tachypnea and hypoxemia. This alignment supports the external validity of our findings. Although ICU patients had a nearly normal median SpO₂ (96%), this low-normal value carries clinical significance when accompanied by tachypnea and chest retractions, indicating limited respiratory reserve.

In previous studies, a hospital stay exceeding 5 or 7 days was considered an indicator of severe RSV-LRTI [20, 21]. Our regression analysis identified three concave sign at admission as an independent risk factor for prolonged hospital stay (OR = 5.242), reflecting severe lung inflammation and gas exchange impairment [22]. Three concave sign reflects increased work of breathing and airway obstruction but does not necessarily indicate hypoxemia; early compensation allows preserved oxygenation despite severe distress. However, preserved oxygenation does not rule out severe disease. This clinical sign should be considered an indicator of increased respiratory effort and disease severity, even in the absence of hypoxemia. Therefore, three concave sign should be interpreted as an early warning sign of impending acute respiratory failure, warranting close monitoring, timely assessment of gas exchange, and early consideration of respiratory support.

For severe pneumonia, we found that elevated PCT, WBC, and LDH were independent risk factors. Previous studies have shown that PCT combined with other inflammatory markers can improve the accuracy of predicting severe RSV pneumonia [23]. PCT is a specific marker of systemic inflammation, and its elevation suggests bacterial coinfection or a virus-induced hyperinflammatory response [24]. A study involving hospitalized children with RSV infection revealed a significant association between elevated PCT levels (> 0.25 ng/mL) and the need for mechanical ventilation (*P* = 0.001) [25]. In our RSV-severe pneumonia cohort, the median PCT value was 0.1 (0.08–0.4) ng/mL, which aligns with reported ranges of 0.2–0.84 ng/dL for PCT in pediatric viral infections [26]. RSV infection itself may cause mild PCT elevation, but to a much lesser extent than bacterial infections. In a cohort of severe RSV pneumonia cases without exclusion of bacterial co-infections, the median PCT was 0.3 (IQR = 0.12–1.2) ng/mL; studies have demonstrated that a serum PCT level above 2.25 ng/mL is more effective for detecting bacterial co-infections [27]. Although systematic screening was performed, exhaustive testing (e.g., blood cultures) was not routine, so undetected bacterial co‑infection cannot be completely ruled out. Therefore, the relatively high PCT values observed at the upper limit of distribution for severe RSV pneumonia cases in this cohort may be attributed to the virus-induced systemic inflammatory response and bacterial co-infection. Although WBC and LDH also reached statistical significance, their odds ratios were close to unity, indicating limited clinical relevance. PCT is the most clinically meaningful biomarker among the three.

This study has several limitations. First, it is a two-center retrospective study with a relatively small sample size, and some outcome risk factor analyses yielded atypical results. For example, baseline differences between centers could not be formally adjusted due to small event numbers, though stratified analyses were consistent. The limited ICU events produced wide confidence intervals; despite using Firth regression, the estimates remain exploratory and require validation in larger cohorts. Second, our imaging-based inclusion criterion requiring LRTI may have excluded mild or upper respiratory infections, but this ensured that our findings apply to hospitalized children with moderate-to-severe RSV-LRTI—the population at greatest risk for poor prognosis. Third, observed inter-center differences in hospitalization metrics reflect institutional roles rather than management differences. Both centers used identical ICU admission criteria and respiratory support protocols. The lower ICU admission rate at the county hospital likely reflects differences in disease severity rather than different thresholds. Additionally, longer-term follow-up is lacking for predicting long-term prognosis and recovery. Future studies should expand sample sizes, conduct multicenter investigations, integrate more clinical data, and use advanced statistics/machine learning to build accurate prediction models for early intervention in pediatric RSV infection.

## Conclusion

This two-center study demonstrated that RSV-LRTI predominantly occurs in children under two years of age and peaks in winter and spring. Higher respiratory rate at admission was independently associated with ICU admission, and the presence of three concave sign was independently associated with prolonged hospital stay. PCT was an independent predictor of severe pneumonia. These inexpensive, easy-to-obtain indicators facilitate early identification of critically ill children and are promising prognostic biomarkers for primary healthcare settings.

## Supplementary Information

Supplementary Material 1.

Supplementary Material 2.

Supplementary Material 3.

## Data Availability

The dataset supporting the conclusions of this article is included within its additional file.

## Abbreviations

RSV: Respiratory syncytial virus
LRTI: Lower respiratory tract infection
ICU: Intensive Care Unit
CRP: C-reactive protein
WBC: White blood cell
ALT: Alanine aminotransferase
AST: Aspartate aminotransferase
LDH: Lactate dehydrogenase
CK: Creatine kinase
L/N: Lymphocyte-to-neutrophil ratio
PCR: Polymerase Chain Reaction
CNY: China Yuan
OR: Odds ratios
CI: Confidence Interval
IQR: Interquartile range
