# Supplementary material for: Clinical characteristics and risk factors for poor prognosis of RSV lower respiratory tract infection: a two-center study
Source: BMC Pediatr. 2026 Aug 10;26:1085. doi: 10.1186/s12887-026-07480-4 (PMC13721514; doi:10.1186/s12887-026-07480-4)
Supplement: Supplementary file 1 [file 12887_2026_7480_MOESM1_ESM.docx]

**Table S1. Age‑specific tachypnea across different outcome groups**

| **Group** | **Tachypnea, n/N (%)** | **P value** |
| --- | --- | --- |
| Overall cohort | 7/257 (2.7%) |  |
| **ICU admission** |  | **<0.001** |
| ICU group | 3/9 (33.3%) |  |
| Non‑ICU group | 4/248 (1.6%) |  |
| **Prolonged hospital stay** |  | 1.000 |
| Prolonged group | 1/33 (3.0%) |  |
| Non‑prolonged group | 6/224 (2.7%) |  |
| **Severe pneumonia** |  | 0.202 |
| Severe pneumonia group | 4/77 (5.2%) |  |
| Mild pneumonia group | 3/180 (1.7%) |  |

**Table S2. Variance inflation factors (VIF) for candidate predictors in the ICU Admission**

| **Variable** | **Variance Inflation Factor (VIF)** |
| --- | --- |
| Tachypnea | 1.143 |
| Oxyhemoglobin saturation | 1.099 |
| Three concave sign | 1.192 |
| PCT | 1.230 |
| CRP | 1.216 |

**Table S3. Firth’s penalized logistic regression for ICU admission (n=257, events=9)**

| Variable | OR | 95% CI | P value |
| --- | --- | --- | --- |
| Tachypnea | 2.978 | 0.338–22.706 | 0.316 |
| Oxyhemoglobin saturation | 0.883 | 0.609–1.290 | 0.501 |
| Three concave sign | 23.314 | 4.461–154.956 | **<0.001** |
| PCT | 1.190 | 0.716–2.391 | 0.501 |
| CRP | 1.022 | 0.996–1.049 | 0.088 |

**Table S4. Variance inflation factors (VIF) for candidate predictors in the prolonged hospital stay analysis**

| **Variable** | **Variance Inflation Factor (VIF)** |
| --- | --- |
| Cough | 1.000 |
| Fever | 1.519 |
| Pre‑admission fever days | 1.518 |
| Respiratory rate | 1.889 |
| Three concave sign | 1.838 |
| CK | 1.028 |

**Table S5. Firth’s penalized logistic regression analysis for predictors of prolonged hospital stay including cough (n=257, events=33)**

| **Variable** | **Adjusted OR** | **95% CI** | **P value** |
| --- | --- | --- | --- |
| Cough | 0.024 | 0.0002–0.276 | **0.002** |
| Fever | 0.600 | 0.219–1.702 | 0.330 |
| Pre-admission fever days | 0.983 | 0.830–1.109 | 0.809 |
| Respiratory rate | 0.989 | 0.916–1.061 | 0.766 |
| Three concave sign | 5.720 | 1.467–22.099 | **0.013** |
| CK | 1.001 | 1.000–1.003 | 0.072 |

Note: The extremely low OR for cough is a residual artifact of near‑complete separation and is not clinically interpretable.

**Table S6. The associations between the primary predictors of each center and the outcomes**

| Predictor | Outcome | Nanjing cohort (n=182) | ***P* value of Nanjing** | Gaoyou cohort (n=75) |
| --- | --- | --- | --- | --- |
| Respiratory rate, median (IQR) | ICU admission | 41.5 (34.8–46.3) | **<0.001** | only 1 case, Respiratory rate=49 |
|  | Non‑ICU admission | 30.0 (28.0–32.0) |  | 28 (24–32) |
| Three concave sign, n (%) | Prolonged hospital stay | 4/15 (26.7%) | **0.006** | 3/47 (6.4%) |
|  | Non‑prolonged hospital stay | 8/167 (4.8%) |  | 5/28 (17.9%) |
| PCT, median (IQR) | Severe pneumonia | 0.13 (0.08–0.42) | **<0.001** | only 1 case, PCT=0.06 |
|  | Mild pneumonia | 0.09 (0.05–0.16) |  | 1.12 (0.07-0.26) |
