# Supplementary material for: Clinical characteristics and risk factors for poor prognosis of RSV lower respiratory tract infection: a two-center study
Source: BMC Pediatr. 2026 Aug 10;26:1085. doi: 10.1186/s12887-026-07480-4 (PMC13721514; doi:10.1186/s12887-026-07480-4)
Supplement: Supplementary file 2 [file 12887_2026_7480_MOESM2_ESM.docx]

**Figure S1. Outcome comparison between the two centers.** A. Hospital stay (d). B. Hospitalization expenses (CNY). C. Cases and proportion of ICU and non-ICU. D. Cases and proportion of prolonged hospital stay and non-prolonged hospital stay. E. Cases and proportion of severe pneumonia and mild pneumonia.

**Figure S2. The number and proportion of symptoms and signs observed in pediatric patients within the cohort.** A. The exclusivity and overlap between the three most common symptoms (fever, cough, wheezing) and two signs (three depressive signs and wheezing sounds). B. The frequency and proportion of the three symptoms (fever, cough, and wheezing).

**Figure S3.** Forest plot of binary multifactorial logistic regression for RSV outcomes. A. Traditional multifactorial logistic regression analysis for ICU admission. B. Traditional multifactorial regression model for prolonged hospital stay. C. Traditional multifactorial regression model for severe pneumonia.
